# Imaging-based prognostic factors in patients undergoing thermal ablation for colorectal liver metastases. A retrospective study on the role of sarcopenia parameters and tumor burden score

**DOI:** 10.2478/raon-2026-0011

**Published:** 2026-02-04

**Authors:** Maximilian Moos, Lena Maria Jacobi, Fabian Stoehr, Paul Steiner, Tobias Bäuerle, Roman Kloeckner, Constantin Scholz, Hauke Lang, Lukas Müller, Felix Hahn

**Affiliations:** Department of Diagnostic and Interventional Radiology, University Medical Center Mainz, Mainz, Germany; Department of Internal Medicine I, University Medical Center Mainz, Mainz, Germany; Institute of Interventional Radiology, University Hospital of Schleswig-Holstein - Campus Lübeck, Lübeck, Germany; Department of General, Visceral and Transplant Surgery, University Medical Center Mainz, Mainz, Germany

**Keywords:** psoas muscle volume index (PMVI), sarcopenia, deep learning segmentation, prognostic imaging biomarkers

## Abstract

**Background:**

To investigate imaging-based prognostic factors in patients who underwent thermal ablation for colorectal liver metastases (CRLM), with a focus on sarcopenia-related body composition parameters and L1-bone-density in comparison to tumor burden score (TBS).

**Patients and methods:**

A retrospective analysis was conducted on patients who received thermal ablation for CRLM at our tertiary care center between 2009 and 2023. CT-derived body composition metrics included the psoas muscle volume index (PMVI), the psoas muscle index (PMI), and L1-bone-density. PMVI was automatically extracted using the open-source deep learning tool TotalSegmentator. Comparisons between 1-year survivors and non-survivors were performed using unpaired t-tests.

**Results:**

A total of 88 patients were included, most had previously undergone hepatic resection (n = 72, 82%). Among sarcopenia-related imaging markers, PMVI showed a significant association with 1-year survival (p = 0.048), with higher PMVI values observed in survivors (mean 113.3 cm^3^/m^3^) compared to non-survivors (mean 101.3 cm^3^/m^3^). No significant differences were observed for L1-density (p = 0.925) or PMI (p = 0.137). Similarly, the TBS was not significantly associated with 1-year survival (p = 0.182).

**Conclusions:**

In our cohort of patients treated with thermal ablation for CRLM, PMVI showed significant association with 1-year survival, which was not observed for conventional tumor burden score or other sarcopenia-related imaging parameters.

## Introduction

Colorectal cancer (CRC) is among the most common malignancies worldwide and a leading cause of cancer-related mortality.^1^ Up to 30% develop colorectal liver metastases (CRLM), which significantly impact overall survival and therapeutic management.^2–6^ While surgical resection remains the gold standard for curative treatment of CRLM, not all patients are eligible due to anatomical constraints or comorbidities.^7^ In this context, thermal ablation techniques such as radiofrequency ablation (RFA) and microwave ablation (MWA) have emerged as minimally invasive alternatives offering favorable outcomes in selected patients.^8–10^ Recent studies demonstrate that thermal ablation is not inferior to surgical resection in terms of oncological outcomes and represents a low-risk alternative in appropriately selected patients.^11^

Nevertheless, survival after thermal ablation remains variable, highlighting the need for reliable prognostic biomarkers to guide clinical decisionmaking. Traditionally, tumor-related characteristics such as size and number of liver metastases have been incorporated into risk stratification tools, including the tumor burden score (TBS), which combines both metrics into a single continuous variable.^12^ While TBS has demonstrated prognostic value in patients undergoing hepatic resection^13^, its applicability in the setting of ablation remains less clearly defined, particularly in cohorts with prior surgical interventions or systemic therapies.

In parallel, recent oncologic imaging research has increasingly focused on body composition parameters derived from cross-sectional imaging as potential prognostic markers. Sarcopenia, the progressive loss of skeletal muscle mass and function, has been shown to adversely affect clinical outcomes in cancer patients, including those with gastrointestinal malignancies.^14,15^ Computed tomography (CT)-based assessment of sarcopenia is typically performed at the level of the third lumbar vertebra (L3), using parameters such as the psoas muscle index (PMI), defined as the cross-sectional muscle area normalized by height squared.^16^ However, emerging evidence suggests that volumetric muscle measurements may offer a more robust and comprehensive representation of skeletal muscle status compared to single-slice assessments.^17,18^

In particular, the psoas muscle volume index (PMVI), which reflects the total iliopsoas muscle volume normalized by patient height cubed, has been proposed as a promising sarcopenia metric. PMVI can be derived through automated segmentation techniques enabled by deep learning tools, allowing for more reproducible and efficient analysis.^19^ Alongside muscle mass, CT-derived bone density, such as average Hounsfield units (HU) measured in the trabecular portion of the L1 vertebral body, has also been investigated as a surrogate for frailty and overall physiological reserve.^20,21^ However, the prognostic significance of these parameters specifically in patients undergoing thermal ablation for CRLM has not yet been fully investigated.

Therefore, the aim of this retrospective study was to assess the prognostic relevance of imagingbased body composition parameters, including PMVI, PMI, and L1 density, in a cohort of patients undergoing thermal ablation for CRLM. These markers were compared with the tumor burden score to evaluate their association with short-term survival outcomes. The hypothesis was that volumetric muscle assessment might offer additional prognostic information beyond conventional tumor metrics, thereby supporting individualized risk stratification in this patient population.

## Patients and methods

### Study design and ethics

This retrospective study was conducted in accordance with the Declaration of Helsinki and was approved by the ethics committee of Rhineland-Palatine (permit number: 15913). Due to the retrospective nature of the study, informed consent was waived.

### Patients

Between 2009 and 2023, 96 patients underwent thermal ablation for colorectal liver metastases (CRLM) at our tertiary care center. In total 88 patients who met the inclusion criteria were identified from our clinical database. The inclusion criteria were as follows: 1) age over 18 years, 2) availability of preinterventional CT for the assessment of sarcopenia parameters, 3) availability of clinical, demographic, and laboratory data at the time of treatment initiation, and 4) Follow-up by our care center for 1 year.

### Data acquisition

All patient data were retrospectively retrieved from our institution’s clinical and radiological information systems (Mesalvo, Freiburg, Germany) and the local picture archiving and communicaion system (Sectra, Linköping, Sweden). Body composition metrics related to sarcopenia were extracted from the pre-interventional CT images.

### Psoas muscle index (PMI)/skeletal muscle index (SMI)

**FIGURE 1.**
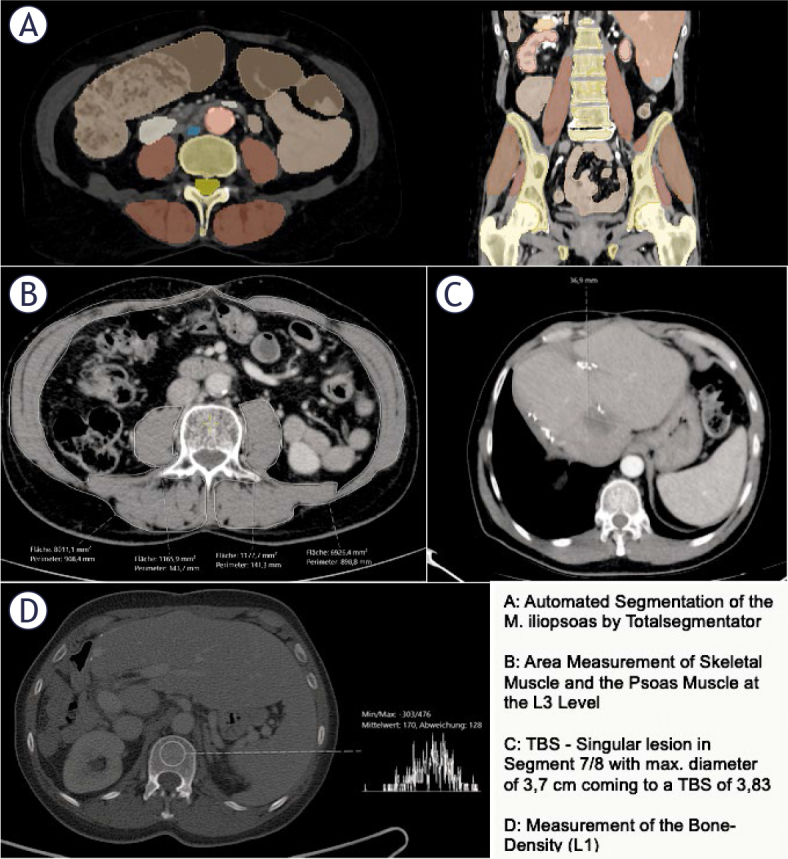
Example of imaging data acquisition of psoas muscle volume index (PMVI), psoas muscle index (PMI), skeletal muscle index (SMI), tumor burden score (TBS) and bone density

To determine the PMI, we used the same pre-interventional images as for the PMVI. Area of the psoas muscle was measured at the level of the third lumbar vertebra using the measurement function of our image archiving system, Sectra, in cm^2^ (Figure 1B). The PMI was then calculated by determining the combined area of the bilateral psoas muscle, divided by the height squared (cm^2^/m^2^). The SMI was measured at the level of L3 corresponding to the PMI (Figure 1B). The SMI was calculated by determining the area of skeletal muscle divided by height squared (cm^2^/m^2^).

### L1-Density

To determine the bone density, a circular region of interest (ROI) was placed in the trabecular bone of the vertebral body of the first lumbar vertebra (L1) (Figure 1, D). The mean CT density (HU) was measured by placing a ROI in the axial slice. During the placement of the ROI, areas that could distort the CT density measurement (such as focal lesions, the internal posterior venous plexus, or imaging-related artifacts) were avoided. The same venous images as those used for the PMVI and PMI determination with a slice thickness of 3.0 mm were used for the analysis. This measurement is often used as an indirect marker of bone quality and can be correlated with overall muscle mass and body composition.^22,23^.

### Tumor burden score (TBS)

To determine the TBS, the number of metastases and the extent of the largest lesion were recorded from the pre-interventional CT (the same as for PMVI, PMI, and L1). TBS was defined using a Cartesian coordinate system, where the maximum tumor diameter (on the x-axis, in cm) and the number of lesions (on the y-axis) were considered. TBS was designed to assess the extent of the liver metastases and their potential impact on patient prognosis. The number of lesions and the maximum diameter of the largest lesion were extracted from the written report of the pre-interventional CT scan and independently verified by a secondyear radiology resident.

### Statistical analysis

All statistical analyses and graphics were performed using R Studio (RStudio Team [2020]. RStudio: Integrated Development for R. RStudio, PBC, http://www.rstudio.com, last accessed May 10, 2025) and R 4.0.3 (A Language and Environment for Statistical Computing, R Foundation for Statistical Computing, http://www.R-project.org). Continuous data were reported as mean ± standard deviation, while categorical and binary parameters were reported as absolute numbers and percentages. The patients were divided into two groups (alive and deceased) over the course of one year, with one-year survival starting on the day of ablation. The variables to be analyzed were PMVI, PMI, SMI, L1 Density, and TBS. Testing for normality of these groups was performed using the Shapiro-Wilk test. Depending on the normality, significance testing to detect differences between the survival groups was conducted using the t-test (for normal distribution) or the Mann-Whitney U test (for non-normal distribution). A correlation analysis of the variables was performed using Spearman’s correlation. Additionally, for each parameter of interest, univariable Cox regression analyses were performed to assess their association with survival. Variables with relevant clinical association or statistical indication were subsequently included in multivariable Cox regression models to adjust for potential confounding factors.

## Results

### Baseline characteristics

The study included 88 patients with a median age of 62 years (IQR: 55–71). Of the patients, 28% were female and 72% were male, with 66% presenting synchronously and 34% metachronously. Most patients (82%) had prior hepatic resection, with a median time of 2 years (IQR: 1–4) from colorectal cancer diagnosis to ablation, a median of 1 CRLM, and a median largest CRLM diameter of 2 cm (IQR: 1.5–2.6). Baseline characteristics are depicted in Table 1.

**TABLE 1.** Baseline characteristics of the cohort

Variable	All patients
Age, years, median (IQR)	62 (55–71)
Sex, n (%)	
Female	25 (28%)
Male	63 (72%)
Temporal presentation n (%)	
Synchronous	58 (66%)
Metachronous	30 (34%)
Prior hepatic resection n (%)	
Yes	72 (82%)
No	16 (18%)
Mean time from CRC diagnosis to ablation, years, median (IQR)	2 (1–4)
Mean CRLM count, median (IQR)	1 (1–1)
Mean diameter of largest CRLM, cm, median (IQR)	2 (1.5–2.6)

1 CRC = colorectal cancer, CRLM = colorectal liver metastases, IQR = interquartile range

**TABLE 2.** Survival groups after thermal ablation

Variable	Alive (first year)	Deceased (first year)
PMVI, cm^3^/m^3^, median (IQR)	113.3 (99.17–129.55)	101.34 (90.93–111.81)
PMI, cm^2^/m^2^, median (IQR)	5.46 (4.6–6.24)	4.74 (4.12–5.46)
SMI, cm^2^/m^2^, median (IQR)	43.78 (40.77–51.96)	40.74 (37.73–48.48)
Ll-density, HU, median (IQR)	134.5 (112–156)	141.5 (103.25–160.5)
Tumor burden score, median (IQR)	2.3 (1.8–3.17)	2.77 (2.19–3.35)

1 HU = Hounsfield units (HU); IQR = interquartile range; PMI = psoas muscle index; PMVI = psoas muscle volume index; SMI = skeletal muscle index

### Survival groups after thermal ablation

**FIGURE 2.**
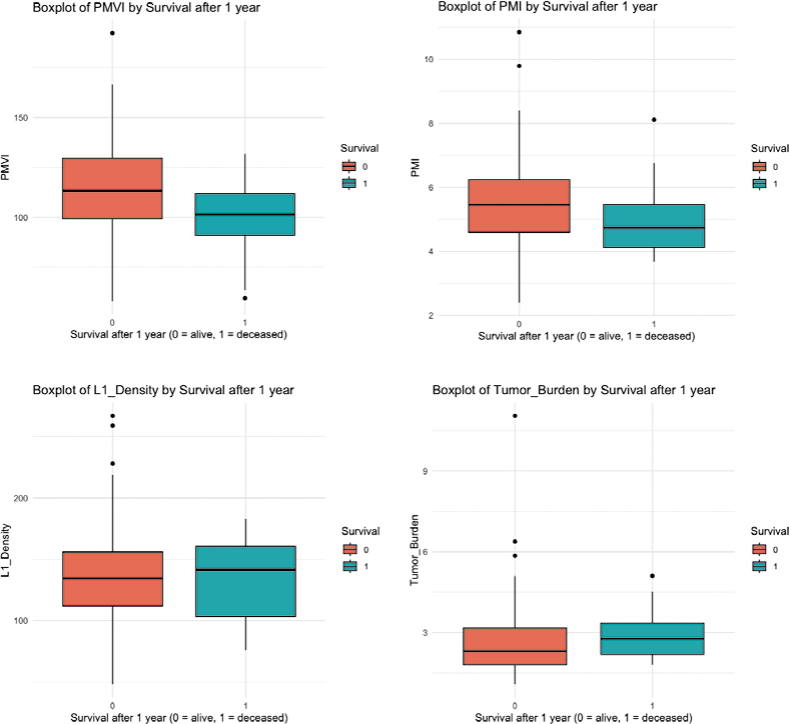
Boxplots of the survival group analysis. PMI = psoas muscle index; PMVI = psoas muscle volume index

For PMVI, one-year-survivors had a median of 113.30 cm^3^/m^3^, while those who died had a median of 101.34 cm^3^/m^3^, with IQRs of 30.38 and 20.87, respectively (Figure 2). In terms of PMI, survivors showed a median of 5.46 cm^2^/m^2^, compared to 4.74 cm^2^/m^2^ for deceased patients, with IQRs of 1.64 and 1.34. For L1-Density, survivors had a median of 134.50 HU, while deceased patients had a median of 141.50 HU, with IQRs of 44.00 and 57.25,% and for TBS, survivors had a median of 2.30, whereas those who died had a median of 2.77, with IQRs of 1.37 and 1.15.

Normality was assessed using the Shapiro-Wilk test. Only PMVI showed normal distribution in both survival groups (p > 0.05). PMI, L1-Density, and TBS showed significant deviations from normality (p ≤ 0.05 in at least one group).

Based on these findings, the following tests were applied:

- For PMVI, an independent samples t-test was performed
- For PMI, SMI, L1-Density, and TBS, the Mann-Whitney U test was applied.

The t-test for PMVI demonstrated a statistically significant difference between survival groups (t = 2.14, p = 0.048, 95% confidence interval: 0.13 to 30.02; means: 114.58 cm^3^/m^3^ vs. 99.50 cm^3^/m^3^) (Table 3).

**TABLE 3.** t-Test/Mann-Whitney-U-Test to show differences in survival groups

Variable	Test	Test Statistic (t/W)	p-Value	Result
PMVI	t-Test (Welch)	t = 2.14	0.048	Significant
PMI	Mann-Whitney U Test	W = 519	0.137	Not significant
SMI	Mann-Whitney U Test	W = 521	0.107	Not significant
L1-Density	Mann-Whitney U Test	W = 415.5	0.925	Not significant
TBS	Mann-Whitney U Test	W = 308.5	0.182	Not significant

1 PMI = psoas muscle index; PMVI = psoas muscle volume index; SMI = skeletal muscle index; TBS = tumor burden score

For PMI, the Mann-Whitney U test showed no statistically significant difference (p = 0.137). Similarly, no significant differences were observed for L1-density (p = 0.925) or TBS (p = 0.182).

In univariable Cox regression analysis, only PMVI (HR 0.97, 95% CI 0.95–1.00; p = 0.049) and age at ablation (HR 1.10 per year, 95% CI 1.03–1.18; p = 0.005) were significantly associated with overall survival (Table 4). In multivariable analysis, age at ablation remained independently associated with survival (HR 1.13, 95% CI 1.03–1.24; p = 0.013), whereas the effect of PMVI was no longer statistically significant (p = 0.51) (Table 4).

**TABLE 4.** Univariate and multivariate Cox regression analysis of baseline clinical, psoas muscle volume index (PMVI), PMI = psoas muscle index (PMI), skeletal muscle index (SMI), L1-density and tumor burden score (TBS)

Covariate	Univariate			Multivariate		
	HR	95% CI	P-value	HR	95% CI	P-value
Age	1.10	1.03–1.18	0.005	1.13	1.03–1.24	0.013
Gender (female)	1.58	0.46–5.39	0.467	3.30	0.56–19.5	0.189
ECOG performance status	1.88	0.5–7.00	0.347	1.39	0.33–5.96	0.654
Temporal presentation CRLM (synchronous)	0.61	0.19–2.00	0.417	1.33	0.30–5.90	0.705
Chemotherapy (yes)	1.2	032–4.52	0.789	2.79	0.56–13.9	0.209
PMVI	0.97	0.95–0.99	0.049	0.99	0.94–1.03	0.509
PMI	0.78	0.51–1.21	0.273	1.53	0.62–3.77	0.358
SMI	0.94	0.87–1.01	0.159	0.94	0.80–1.10	0.417
L1 -density	1.00	0.98–1.01	0.502	1.01	0.99–1.03	0.487
TBS	1.13	0.83–1.56	0.440	1.42	0.91–2.23	0.124

1 CRLM = colorectal liver metastases; ECOG= Eastern Cooperative Oncology Group; PMI = psoas muscle index; PMVI = psoas muscle volume index; SMI = skeletal muscle index; TBS = tumor burden score

### Correlation between the variables

**FIGURE 3.**
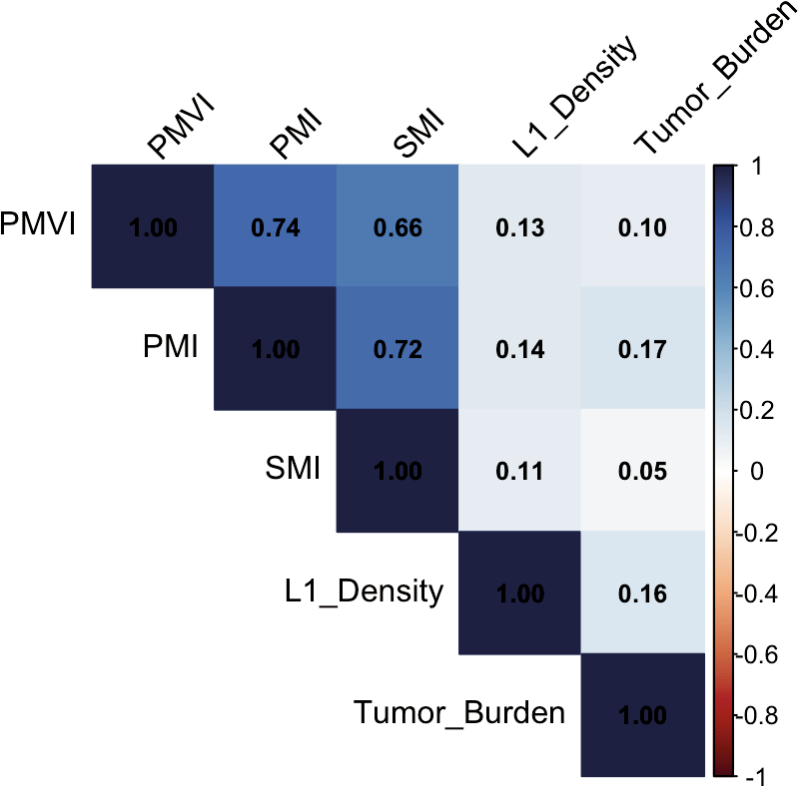
Heatmap of the correlation between the variables.

In addition, a Spearman rank correlation analysis was performed to assess potential associations between the variables PMVI, PMI, SMI, L1-Density, and TBS (Figure 3). A strong positive correlation was observed between PMVI, SMI and PMI. All other correlations were weak, with Spearman’s rho values below 0.2.

PMI = psoas muscle index; PMVI = psoas muscle volume index; SMI = skeletal muscle index

## Discussion

In this retrospective analysis of patients treated with thermal ablation for CRLM, PMVI emerged as a significant imaging biomarker associated with 1-year survival. In contrast, traditional prognostic markers such as the TBS, PMI, and L1-density did not show significant associations with survival outcomes.

The lack of prognostic value of the TBS in our cohort warrants closer examination, especially given that the TBS has been validated as a predictor of survival in resectable and unresectable liver metastases. An explanation is the higher TBS values in other studies, reaching up to 20^12,13,24^, whereas the interquartile range of the TBS in our study was narrow (1.89–3.17). This limited variability likely reflects a selection bias inherent to the ablation setting, where patients with higher tumor burden are typically excluded from this modality in favor of resection or systemic therapy. Most patients in our study had no more than two lesions, consistent with established indications for thermal ablation^25^, which may explain the reduced prognostic utility of the TBS in this context.

The PMI has been evaluated as a prognostic factor in various malignancies, including CRLM. Peng *et al*. identified a significant association between low PMI and poor survival as well as postop complications in patients with liver metastases from colorectal cancer undergoing hepatic resection.^26^ Additionally, Imai *et al*. explored the role of the PMI as an independent predictor of survival in patients with HCC undergoing systemic therapy.^27^ These studies underscore the potential of PMI as a prognostic marker across a range of malignancies, particularly in those with hepatic metastasis or primary liver tumors, and in those receiving diverse therapeutic interventions.

Kamada *et al*. identified bone density as a superior prognostic marker compared to the PMI for predicting survival in patients with CRC.^28^ In contrast, our study, which focused on patients with CRLM, found no significant association between either L1 bone density or PMI with survival outcomes. An explanation for this discrepancy could be the smaller patient cohort in our study, which may have limited the statistical power of our analysis, especially with regard to PMI. Additionally, Kamada *et al*. investigated CRC while our study specifically examined the subgroup of CRLM. This and the different therapies could have contributed to variations in the prognostic relevance of these factors.

Among the parameters investigated in our study, only the PMI and psoas muscle volume index PMVI exhibited a significant correlation (r = 0.72). This correlation is consistent with the findings of Manabe *et al*. (2023), who also observed a strong association between PMI and PMVI (r = 0,85) in patients with liver disease.^18^ In their study, the authors emphasized the usefulness of PMVI in diagnosing sarcopenia and its relationship with skeletal muscle mass.

Notably, among the sarcopenia-related parameters, only the PMVI was significantly associated with survival. The superiority of the PMVI over the PMI is likely attributable to its volumetric approach, which captures the full bilateral iliopsoas muscle mass, as opposed to the PMI, which relies on a single axial cross-sectional measurement at the L3 level. Several studies have shown that axial measurements of muscles/nodules may lead to inaccurate results compared to volumetric assessments.^29,30^ Measurements at the level of L3 offer a range of possible values (depending on the exact slice location within the vertebral body), which may result in different outcomes for the same patient. Thus, more comprehensive volumetric assessment may better reflect overall muscularity and functional reserve in oncologic patients. Additionally, the automated extraction of PMVI using an open-source deep learning tool (TotalSegmentator) enhances reproducibility and feasibility in clinical workflows.^19^

Our findings support the incorporation of volumetric muscle assessment into pre-interventional imaging protocols. Given that sarcopenia has been linked to poor outcomes in multiple cancer types and therapeutic settings^31^, PMVI could serve as a practical and non-invasive tool for risk stratification in patients undergoing local ablative therapies. While PMVI may not be a sole deciding factor for ablation eligibility, it could provide an additional consideration in the decision-making process, potentially leading to future integrative approaches such as pre-therapy exercise or nutritional interventions.

Our study has several limitations. A limitation of this single center study is the small cohort size (n = 88), which may limit the generalizability of the results despite the statistically significant findings observed. Another key aspect of our study is the clinical heterogeneity of the patient cohort. While all patients received thermal ablation, many had undergone prior hepatic resections and were at different stages of systemic treatment. This inhomogeneity could be seen as a limitation; however, it may also underscore the robustness of PMVI as a prognostic marker. Despite the varying therapeutic trajectories, PMVI maintained a statistically significant association with survival, suggesting its potential utility across diverse clinical scenarios.
